# Editorial: Influence of intimate partner violence and male partner involvement in maternity care in low-and-middle income countries

**DOI:** 10.3389/fgwh.2024.1513159

**Published:** 2024-11-18

**Authors:** Guy-Lucien Whembolua, Daudet Ilunga Tshiswaka, Adi Chereni

**Affiliations:** ^1^ Department of Africana Studies, University of Cincinnati, Cincinnati, OH, United States; ^2^ Department of Public Health, University of West Florida, Pensacola, FL, United States; ^3^ School of Social Sciences and Professions, London Metropolitan University, London, United Kingdom; ^4^ Center for Academic Technologies, University of Johannesburg, Johannesburg, South Africa

**Keywords:** maternity care, intimate partner violence, low-and-middle income countries, male partner, influence

**Editorial on the Research Topic**
Influence of intimate partner violence and male partner involvement in maternity care in low-and-middle income countries

In this issue of *Frontiers in Global Women's Health*, we present papers that focus on the influence of Intimate Partner Violence (IPV) and male partner involvement in maternity care in low-and-middle income countries.

Since the mid-1990s, attention has increasingly shifted toward investigating and promoting men's involvement in maternal and child health (1). Numerous studies have highlighted the positive impact of male partner's involvement on maternal health care services. Specifically, male partner involvement in maternal care reduces risk of HIV transmission to infants of HIV-positive mothers (2), improves adherence to recommended infant feeding practices (3), and increases uptake of maternal health services such as antenatal care (4). This involvement is also linked to reduce risks of preterm births, low birth weight, fetal growth restrictions, and infant mortality (5–7). Conversely, extensive research has shown that IPV negatively influences maternal health outcomes, with affected women facing higher rates of physical trauma, suicidal ideation, and increased visits to emergency rooms (8–13).

Globally, women's utilization of maternity health care services has been shown to improve women's health outcomes. In low-and-middle income countries from which the articles in this issue draw, effective use of maternal health services has been reported to reduce maternal mortality and both short- and long-term morbidity, with positive impacts on women's overall health and well-being during pregnancy, postpartum, and beyond (14). More specifically, this special issue highlights various aspects of male partner involvement in women's maternity care, revealing that, in low-and middle-income countries, men also play a significant role in encouraging greater use of maternity care services (15), or, conversely, they prevent maternity care access due to IPV, ultimately impacting women's health outcomes.

A first thread connecting the articles in this issue is the role of partner involvement and factors influencing their engagement in women's health care use. Through a community cross-sectional study in Ethiopia, Aman et al. studied husbands' intent to support the use of maternity waiting home. They found that husbands who adhered to specific cultural norms and felt in control of barriers were more likely to plan on supporting their partner's use of a maternity waiting home. Similarly, in Nigeria, Akinyemi and Ibrahim conducted a mixed-methods study to examine the predictors of men's involvement in pregnancy care. Overall, they found that unmarried men were significantly less likely to be involved in pregnancy care compared to their married counterparts. Similarly, men with negative perceptions toward supporting their partners in antenatal visits had lower odds of involvement in pregnancy care than those with positive perceptions.

Conversely, as illustrated in our second group of articles, IPV is associated with a lower likelihood of using maternity care (16). To study this issue, Adetutu et al. further explored the influence of IPV and male involvement in the utilization of maternal care services by analyzing data from the 2018 Nigeria Demographic and Health Survey (NDHS). Interestingly, they found that women who experienced sexual violence were more likely to use heath facility for antenatal care. In Kenya, a country that has an estimated lifetime prevalence of IPV in women to 38% (17), Schellhammer et al. analyzed household surveys conducted in six wards of Migori County, focusing on identifying factors associated with IPV and assessing its effects on maternal care, based on responses from female participants. They found that having experienced IPV was negatively associated with attending at least four antenatal care visits during the most recent pregnancy and with having a skilled birth attendant.

Thirdly, IPV has a strong negative impact on maternal and infant health outcomes. More specifically, in Brazil, Blumrich et al. examined data from two birth cohorts in two distinct cities collected during pregnancy and at the beginning of the second year of life. They reported an association of violence during pregnancy with infant morbidity in a poorer socioeconomic setting. Meanwhile, in their work in Ghana, Okoror et al. focused on financial abuse experienced by nursing mothers from their significant others. Through thematic analysis, they highlight that nursing mothers who reported lack of financial support perceive it as hinderance to their efforts to care for their children.

Lastly, Alemu et al. described the factors contributing to IPV among women during the recent COVID-19 pandemic. Their review of the literature indicated that having a partner who has either a history of alcohol use or no formal education, women who had lost income during COVID-19, and household decisions made by the husband alone were significant factors for intimate partner violence during the COVID-19 pandemic.

In summary, these papers collectively highlight the need for a better understanding of the different dimensions linked to male involvement in maternity and infant care particularly in low-and-middle income countries. While enhancing men's involvement in maternity and infant care may require community-based awareness, future interventions should: (1) address men's socio-cultural beliefs to improve their participation, (2) address social and gender norms that perpetuate IPV, (3) provide women who are victims of violence with comprehensive healthcare. These actionable recommendations presented by the authors may prove to be difficult to implement but offer a pathway toward social justice for activists and health practitioners in resource limited environments.
